# Total C-21 Steroidal Glycosides From *Baishouwu* Ameliorate Hepatic and Renal Fibrosis by Regulating IL-1β/MyD88 Inflammation Signaling

**DOI:** 10.3389/fphar.2021.775730

**Published:** 2021-10-26

**Authors:** Tingting Qin, Mingliang Wang, Ting Zhang, Yingyu Wang, Yunyun Zhang, Muhammad Hasnat, Zirui Zhuang, Yongfang Ding, Yunru Peng

**Affiliations:** ^1^ Affiliated Hospital of Integrated Traditional Chinese and Western Medicine, Nanjing University of Chinese Medicine, Nanjing, China; ^2^ Department of Pharmacology and Toxicology, Jiangsu Province Academy of Traditional Chinese Medicine, Nanjing, China; ^3^ Institute of Pharmaceutical Sciences, University of Veterinary and Animal Sciences, Lahore, Pakistan

**Keywords:** hepatic fibrosis, renal fibrosis, inflammation, total C-21 steroidal glycosides, IL-1β/MyD88 signaling

## Abstract

Fibrosis is a worldwide public health problem, which typically results from chronic diseases and often leads to organ malfunction. Chronic inflammation has been suggested to be the major trigger for fibrogenesis, yet mechanisms by which inflammatory signals drive fibrogenesis have not been fully elucidated. Total C-21 steroidal glycosides (TCSG) from *Baishouwu* are the main active components of the root of *Cynanchum auriculatum* Royle ex Wight, which exert hepatoprotective and anti-inflammation properties. In this study, we established a mouse model with the coexistence of hepatic and renal fibrosis and aimed to investigate the effects of TCSG from *Baishouwu* on fibrosis and explored the potential mechanisms. The results of biochemical and pathological examinations showed that TCSG from *Baishouwu* improved liver and kidney function and alleviated hepatic and renal fibrosis by reducing collagen and extracellular matrix deposition in bile duct ligation and unilateral ureteral occlusion (BDL&UUO) mice. According to network pharmacology analysis, the mechanisms underlying the effects of TCSG from *Baishouwu* on hepatic and renal fibrosis were associated with inflammatory response pathways, including “Signaling by interleukins”, “MAP kinase activation”, “MyD88 cascade initiated on plasma membrane”, and “Interleukin-1 family signaling”. Regression analysis and western blot results revealed that IL-1β/MyD88 inflammation signaling played an essential role in the anti-fibrotic effects of TCSG from *Baishouwu*. Further data displayed that TCSG from *Baishouwu* affected inflammatory response and extracellular matrix deposition via suppressing the activation of p38 MAPK/JNK and NF-κB p65 signaling cascades both in the liver and kidney of BDL&UUO mice. Thus, our findings suggest TCSG from *Baishouwu* as a natural regimen against hepatic and renal fibrosis and provide direct evidence that IL-1β/MyD88 signaling crucially contributes to hepatic and renal fibrosis and modulates liver-kidney crosstalk by maintaining tight control over inflammatory responses.

## Introduction

Fibrosis is a pathological response characterized by abnormal hyperplasia and excessive deposition of extracellular matrix (ECM) during the process of repair after tissue damage, which has been considered to account for up to 45% of all deaths in the industrialized world (Distler and Györfi, 2019; Henderson et al., 2020). During the pathological process of a variety of chronic diseases, excessively accumulated ECM results in fibrogenesis and increases tissue hardness, which subsequently blocks the diffusion of oxygen and nutrients, further damaging the organs, particularly, hepatic and renal fibrosis are commonly detected in clinical practice (Weiskirchen et al., 2019; Zhao et al., 2020). Emerging studies demonstrate that hepatic and renal fibrosis share similar pathogenesis and pathological characteristics, showing a potential interaction in the pathogenesis, although they occur in different organs. Liver cirrhosis and non-alcoholic fatty liver disease are suggested as independent risk factors for the incidence of chronic kidney disease (Targher et al., 2011; Bernardi et al., 2015). Alternatively, increasing pieces of evidence establish that chronic kidney disease can lead to liver dysfunction, contribute to the development of non-alcoholic fatty liver disease, and associate metabolic disturbances (Musso et al., 2015; Marcuccilli and Chonchol, 2016). These findings drove a popular area of scientific interest in liver-kidney organ crosstalk during the last decade. However, the related pathogenetic mechanisms are not fully known that it is necessary to elucidate the complex and intertwined mechanisms linking hepatic and renal fibrosis and develop effective antifibrotic therapeutic strategies.

Many distinct factors have been revealed to participate in the progression of fibrotic diseases, among which chronic inflammation is thought to be the major contributing factor. It is now clear that persistent inflammation resulting in a chronic wound-healing response can facilitate an increase in the deposition of ECM and sustained tissue damage both in the liver and kidney, ultimately leading to fibrosis (Heymann and Tacke, 2016; Tecklenborg et al., 2018). Consequently, focusing on the cellular mechanisms involved in the stimulation of inflammation and identifying the key mediators have been considered as the targets to alleviate hepatic and renal fibrosis. Traditional Chinese medicine (TCM), widely used in Asian countries for thousands of years, possesses a dual role in immunological regulation, as well as anti-fibrotic actions in the liver and kidney (Ma et al., 2013; Zhang and Schuppan, 2014; Shen et al., 2018; Wang et al., 2020). The root of *Cynanchum auriculatum* Royle ex Wight is a famous TCM known as *Baishouwu*, which has been used as a tonic medicine or healthy food for centuries. Based on the theory of TCM, this herb exhibits medicinal properties, including nourishing the liver and kidney and enhancing immune function. Pharmacological studies reveal that *Baishouwu* shows remarkable hepatoprotective, anti-inflammatory, and antitumor bioactivities (Jang et al., 2016; Li et al., 2018; Chen et al., 2019). The total C-21 steroidal glycosides (TCSG) are commonly accepted as one of the most bioactive ingredients separated from *Baishouwu* (Chen et al., 2019). Previously, we reported that TCSG from *Baishouwu* protected the liver against the damage of oxidative toxicity and inflammatory reactions (Cui et al., 2019; Zhang et al., 2021). We also found that TCSG from *Baishouwu* inhibited the development of hepatocellular carcinoma by suppressing the hepatic inflammation-fibrosis axis induced by diethylnitrosamine (Ding et al., 2017; Ding et al., 2019). Moreover, our recent research demonstrated that TCSG from *Baishouwu* relieved renal fibrosis by regulating the process of renal tubular epithelial-mesenchymal transformation (Yin and Peng, 2020). However, it is not yet clear whether TCSG from *Baishouwu* can regulate hepatic and renal fibrosis, or the involved regulation procession and molecular mechanisms.

In the present study, we aimed to evaluate whether TCSG from *Baishouwu* had therapeutic effects on hepatic and renal fibrosis in the mouse model of bile duct ligation and unilateral ureteral occlusion (BDL&UUO) with a parallel focus on the role of inflammation in the liver-kidney axis. To further explore the underlying mechanism, system network pharmacology analysis was applied to predict the potential mechanism network and then validation experiments were carried out.

## Materials and Methods

### Animals

Adult male C57BL/6 mice weighing 20–22 g were purchased from Shanghai Slack Laboratory Animal Co., Ltd (Shanghai, China). Animal experiments were performed in accordance with the Guidelines for the Care and Use of Laboratory Animals published by the US National Institutes of Health (Publication No.85-23, revised 1996). The care, handling, and experimental procedures of animals were approved by the Ethics Committee of Jiangsu Integrated Traditional Chinese and Western Medicine Hospital (NO. AEWC-20180712-37). Mice were housed under a 12 h light/dark cycle at 22–25°C with 50–60% humidity and provided access to food and water *ad libitum*. All mice were subjected to a 1-week acclimatization period prior to the experimental protocol.

### Animal Surgery and Experimental Design

Mice were randomly allocated into the sham group (*n* = 10) and model group (*n* = 45). The mouse model of hepatic and renal fibrosis was established by BDL and UUO surgical operation under sterile conditions according to previous research (Chevalier et al., 2009; Yokota et al., 2018). Briefly, experimental mice were anesthetized with isoflurane. The experimental area was disinfected with 75% alcohol. After the application of antiseptic on the skin, a 2–3 cm incision was made just below the xiphoid process. The common bile duct was identified, ligated, and two surgical knots were fixed. Then a second bile duct ligation was added in the same manner without division to avoid any interference caused by the loose end. Meanwhile, the ureter was identified, ligated, but not divided as described in the bile duct surgery. The sham group underwent a sham procedure, in which the bile duct and ureter were identified and exposed without ligation. 24 hours after surgery, animals subjected to the model group were randomly divided into three groups (*n* = 15 per group): the model group, the TCSG low-dose group (180 mg/kg/day, TCSG-L), and the TCSG high-dose group (360 mg/kg/day, TCSG-H), and received intragastric drug treatment every other day for 14 days. The extract of TCSG from *Baishouwu* was performed as described in our previous study (Ding et al., 2019). The sham group and model group were received the same volume of vehicles. At the end of the experiment, blood samples were collected from the retroorbital venous plexus. Then animals were sacrificed under isoflurane anesthesia, followed by liver and kidney samples collected for further analysis.

### Serum and Urine Biochemistry

Blood samples were centrifuged (3,000 g, 10 min, 4°C) to prepare serum. Urine samples were diluted using ice-cooled normal saline and centrifuged (5,000 g, 5 min, 4°C) to prepare the clear supernatant. An automatic analyzer (C8000 Roche, Hoffmann-La Roche Inc, Switzerland) and commercial kits (Nanjing Jiancheng Bioengineering Institute, Nanjing, China) were employed to analyze serum and urine biochemistry, including serum levels of alanine transaminase (ALT), aspartate transaminase (AST), total bilirubin (T-Bil), alkaline phosphatase (ALP), total cholesterol (T-CHO), albumin and globulin ratio (A/G), blood urea nitrogen (BUN), and creatinine (Cre), as well as the content of urine protein and urine Cre.

### Histological Analysis

Liver and kidney tissues were fixed in paraformaldehyde solution followed by paraffin embedding. Tissue sections with 5 μm thickness were stained with hematoxylin and eosin (HE) to assess inflammation and necrosis. The Masson trichrome staining was used to evaluate liver and kidney fibrotic alterations. Immunohistochemistry was applied using paraffin sections incubated with primary antibodies: α-SMA (1:100, #19245, Cell Signaling Technology) and TGF-β1 (1:100, #92486, Abcam). Images were observed using a Leica microsystem (DMi8, Leica Microsystems, German, Weztlar). The percentage of the stained positive area was quantified by ImageJ software.

### The Level of Tissue Hydroxyproline

Tissue hydroxyproline (Hyp) was assessed to investigate the level of collagen deposition, which is an index of liver and kidney fibrosis (García et al., 2002). The content of Hyp in tissues was determined using commercial kits (Nanjing Jiancheng Bioengineering Institute) according to the instructions. Generally, the tissue slices were digested in 1 ml hydrochloric acid (6 mol/l) for 5 h under 100°C. After cooling, a 10 μl indicator was added following the addition of solutions A and B for making the solution PH = 6. Then the mixture was centrifugated (3,500 g, 10 min) and the supernatant was incubated with the other test solutions at 60°C for 15 min. Centrifugation was performed (3,500 g, 10 min) and the absorbance of samples was measure at 550 nm using the microplate reader (NanoQuant, Switzerland).

### Network Pharmacology

The network pharmacology technique was used to explore the underlying mechanisms of TCSG in the treatment of hepatic and renal fibrosis. There are 12 active ingredients identified in TCSG from *Baishouwu* according to the literature and our previous study by LC-Q/TOF-MS (Wang et al., 2017; Chen et al., 2019). The details of the twelve active ingredients were shown in Supplementary Figure S1. The targets of TCSG active ingredients were identified by searching the genes database of Swiss Target Prediction database (http://www.swisstargetprediction.ch/index.php) and Pubmed (https://pubmed.ncbi.nlm.nih.gov/). The proven targets for hepatic fibrosis and renal fibrosis were identified by searching DisGeNet database (https://www.disgenet.org/). There were 1,179 target genes related to hepatic fibrosis and 570 target genes associated with renal fibrosis which were consolidated in Excel. The potential therapeutic targets were collected from the interactions of target genes and the networks were constructed by using Cytoscape 3.8.0. The STRING database (https://www.string-db.org/) was used to obtain the interaction relationship between target genes.

### Protein-Protein (PPI) Interaction Network

PPI networks were constructed to investigate the molecular mechanisms of TCSG intervening hepatic and renal fibrosis. TCSG-disease PPI networks were constructed by merging the TCSG network and the disease network. The Network Analyzer plug-in was used to calculate the networks node parameters and the module analysis was carried out by the MCODE plug-in to screen potential therapeutic targets.

### Pathway and Functional Enrichment Analysis

The underlying pathways associated with TCSG against fibrosis of the liver and kidney were analyzed by the ClueGO plug-in. The gene ontology (GO) database (http://geneontology.org/) was applied for function enrichment analysis and the Reactome Pathway database (https://reactome.org/) was used for pathway enrichment analysis.

### Quantitative Real-Time PCR

Total RNA of liver and kidney tissues was extracted with TRIzol (Thermo Fisher Scientific, Shanghai, China) according to the manufacture’s protocol. RNA was reversely transcribed into cDNA and quantitative real-time PCR (qRT-PCR) was performed on the detection systems (Bio-Rad, United States). Primer sequences used in the study were shown in Table 1. Samples were initially denaturized at 95°C for 15 min and processed under 40 consecutive thermal cycles (94°C, 15 s; 60°C, 30 s; 72°C, 30 s) followed by the final melting-curve cycle. The relative abundance was calculated by the ∆∆Ct method. The transcript levels of genes were normalized to GAPDH in the corresponding sample.

**TABLE 1:** PCR primer sequences.

Target gene	Forward primer (5–3′)	Reverse primer (5–3′)
GAPDH	TGC ACC ACC AAC TGC TTA G	GGA TGC AGG GAT GAT GTT C
TGF-β1	CTT CAA TAC GTC AGA CAT TCG GG	GTA ACG CCA GGA ATT GTT GCT A
α-SMA	TCC CTG GAG AAG AGC TAC GAA CT	AAG CGT TCG TTT CCA ATG GT
IL-1β	TGG​ACC​TTC​CAG​GAT​GAG​GAC​A	GTT​CAT​CTC​GGA​GCC​TGT​AGT​G
Col 1a1	CCT GGC AAA GAC GGA CTC AAC	GCT GAA GTC ATA ACC GCC ACT G
Col 3a1	CTG TAA CAT GGA AAC TGG GGA AA	CCA TAG CTG AAC TGA AAA CCA CC

### Western Blot Analysis

The total proteins from liver and kidney tissues were lysed by RIPA buffer (Beyotime Biotechnology, Shanghai, China) containing 1% PMSF. Protein concentrations were determined using a BCA protein assay kit (KeyGEN Biotech, Nanjing, China). The equal amount of lysates (30 μg) were separated by SDS-PAGE and then electrophoretically transferred to PVDF membranes (Millipore Billerica, MA, United States). Membranes were blocked with 5% skim milk in TBST at room temperature for 1 h and then incubated with primary antibodies, MyD88 (1:500, ab2064, Abcam), TRAF6 (1:2000, ab33915, Abcam), IKKα (1:1,000, ab32518, Abcam), p-IKKα (1:1,000, #2859, Cell Signaling Technology), NF-kB p65 (1:1,000, #8242, Cell Signaling Technology), p38 (1:1,000, #8690, Cell Signaling Technology), p-p38 (1:1,000, #4511, Cell Signaling Technology), JNK (1:1,000, #9252, Cell Signaling Technology), p-JNK (1:1,000, #4668, Cell Signaling Technology), Lamin B (1:1,000, ab133741, Abcam), and GAPDH (1:1,000, #2118, Cell Signaling Technology), overnight at 4°C. HRP-conjugated anti-rabbit/mouse IgG was then incubated with membranes for 1 h at room temperature. The blots were imaged using electrochemiluminescence with Tanon Automatic Chemiluminescence Image analysis system. The quantitative analysis was determined using Image J software.

### Statistical Analysis

All data were expressed as mean ± SEM. GraphPad Prism 8 software was used for statistical analysis. Statistical significance was determined by using one-way analysis of variance (ANOVA) with Turkey’s post hoc test. The significant difference was considered at *p* < 0.05.

## Results

### TCSG From *Baishouwu* Ameliorated Liver Damage in BDL&UUO Mice

**FIGURE 1:**
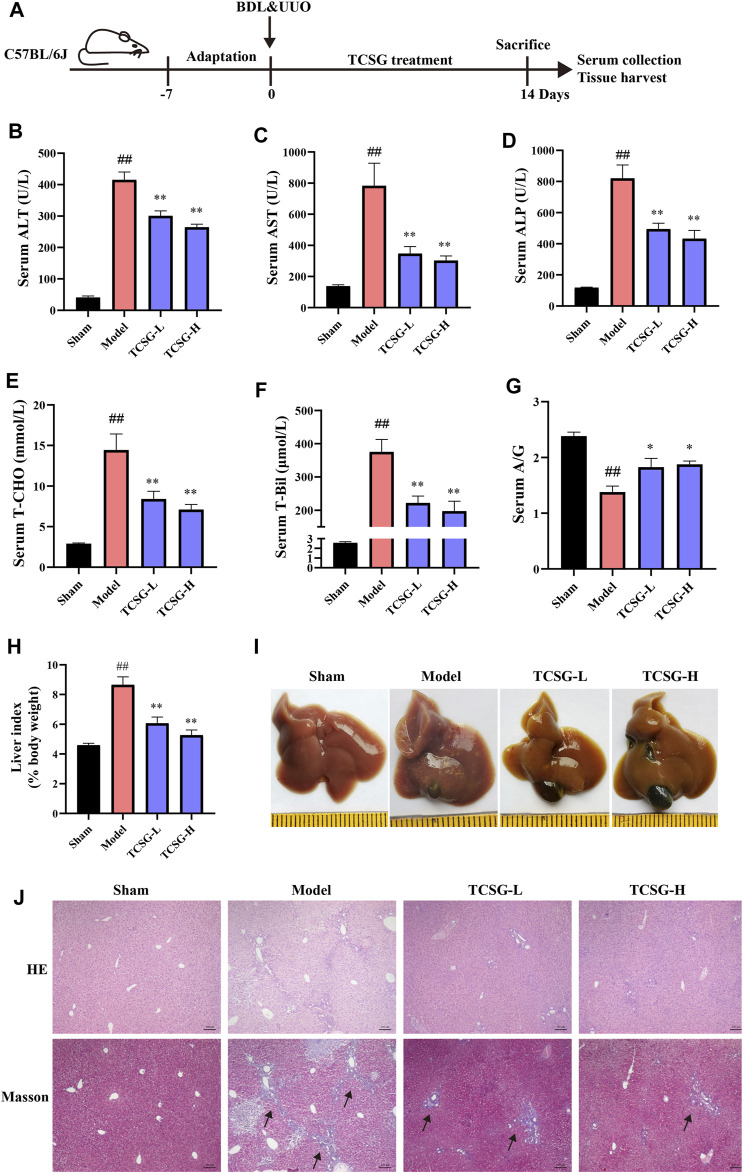
TCSG from *Baishouwu* ameliorated liver damage in BDL&UUO mice. **(A)** Schematic diagram of hepatic and renal fibrosis model with TCSG treatment. After BDL&UUO operation, mice were treated with TCSG for consecutive 14 days, followed by sacrifice and tissues collection. **(B–G)** The effects of TCSG on serum biochemical measurements of liver injury. **(H)** The effects of TCSG on the liver index. **(G)** The effects of TCSG on the morphologic changes of the liver. **(J)** The effects of TCSG on liver pathological changes detected by HE staining and liver collagen accumulation determined by Masson staining (scale bar 100 μm). Data are shown as means ± SEM (*n* = 10–12), ^##^ *p* < 0.01, significantly different from sham group; ^*^ *p* < 0.05, ^**^ *p* < 0.01, significantly different from model group.

To evaluate the effects of TCSG from *Baishouwu* on liver damage in the BDL&UUO mice model, the liver biochemical parameters were firstly detected. Results showed that the levels of serum ALT and AST were significantly higher indicating liver injury in the model mice (Figures 1B,C). Significant elevation in the serum levels of ALP suggested cholestasis in the model mice (Figure 1D). On the other hand, the changes in serum levels of liver function biomarkers were also evident. Serum T-CHO and T-Bil were increased and serum A/G was decreased (Figures 1E–G). It was worth noting that these changes of biochemical markers were improved after the administration of TCSG. Liver tissue histopathological changes were also assessed in the BDL&UUO mice model. Results of the liver index indicated significant hepatomegaly in the model mice (Figure 1H). The liver morphology showed apparent changes in the group of model mice, the surface of which contained many visible white particles (Figure 1I). Further, HE staining and Masson staining revealed the evident inflammation, tissue necrosis, and collagen deposition in the model group (Figure 1J). Nevertheless, treatment with TCSG positively affected the liver index of the model mice, and TCSG administration also improved the liver pathological changes. Together, these data indicated that TCSG from *Baishouwu* attenuated liver damage in the BDL&UUO mice model.

### TCSG From *Baishouwu* Relieved Kidney Injury in BDL&UUO Mice

**FIGURE 2:**
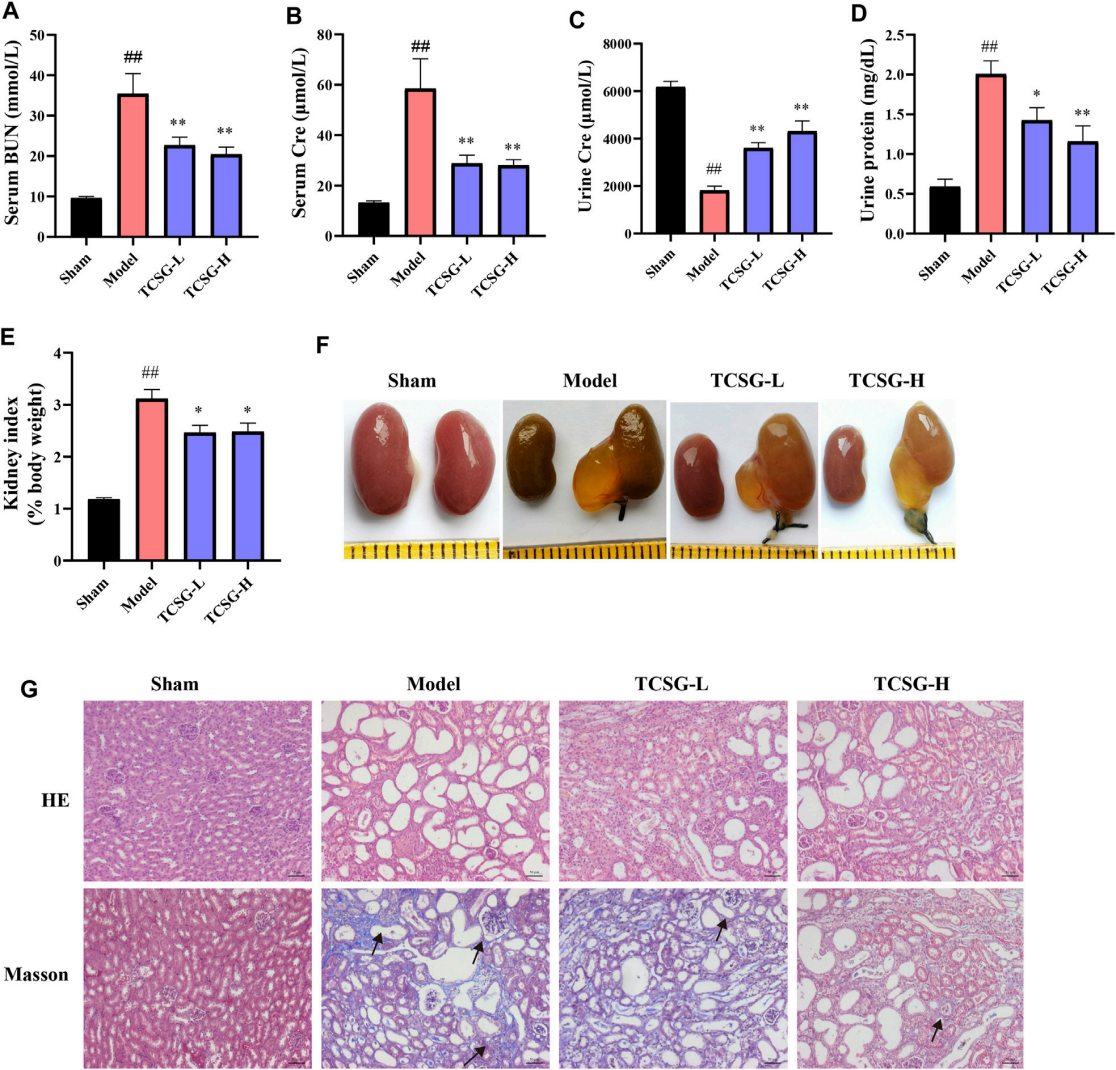
TCSG from *Baishouwu* relieved kidney injury in BDL&UUO mice. **(A,B)** The effects of TCSG on serum biochemical measurements of kidney injury. **(C,D)** The effects of TCSG on urine biochemical measurements of kidney injury. **(E)** The effects of TCSG on the kidney index. **(F)** The effects of TCSG on the morphologic changes of the kidney. **(G)** The effects of TCSG on kidney pathological changes detected by HE staining and kidney collagen accumulation determined by Masson staining (scale bar 50 μm). Data are shown as means ± SEM (*n* = 10–12), ^##^ *p* < 0.01, significantly different from sham group; ^*^ *p* < 0.05, ^**^ *p* < 0.01, significantly different from model group.

Next, the kidney biochemical parameters were investigated to assess the effects of TCSG from *Baishouwu* on kidney injury in the BDL&UUO mice model. As shown in Figure 2, serum BUN and Cre were significantly upregulated in the model group (Figures 2A,B). Urinalysis of biomarkers also revealed an increase in urine protein and Cre (Figures 2C,D). TCSG treatment, both at low and high doses, markedly decreased serum and urine biomarkers, suggesting the alleviated kidney injury in BDL&UUO mice. Moreover, organ weight indices exhibited an abnormal weight of the kidney in the model mice (Figure 2E), and kidney morphology showed partial renal tubular dilatation and vacuolar deformation in the model mice (Figure 2F). HE staining of the model mice presented interstitial inflammation, tubular atrophy, and necrosis meanwhile, Masson staining revealed the obvious collagen deposition (Figure 2G). It was found that not only the organ weight indices but also pathological changes of the kidney showed a corresponding improvement after treatment with TCSG in BDL&UUO mice. These findings suggested that TCSG from *Baishouwu* relieved kidney injury in the BDL&UUO mice model.

### TCSG From *Baishouwu* Inhibited Hepatic and Renal Fibrosis in BDL&UUO Mice

**FIGURE 3:**
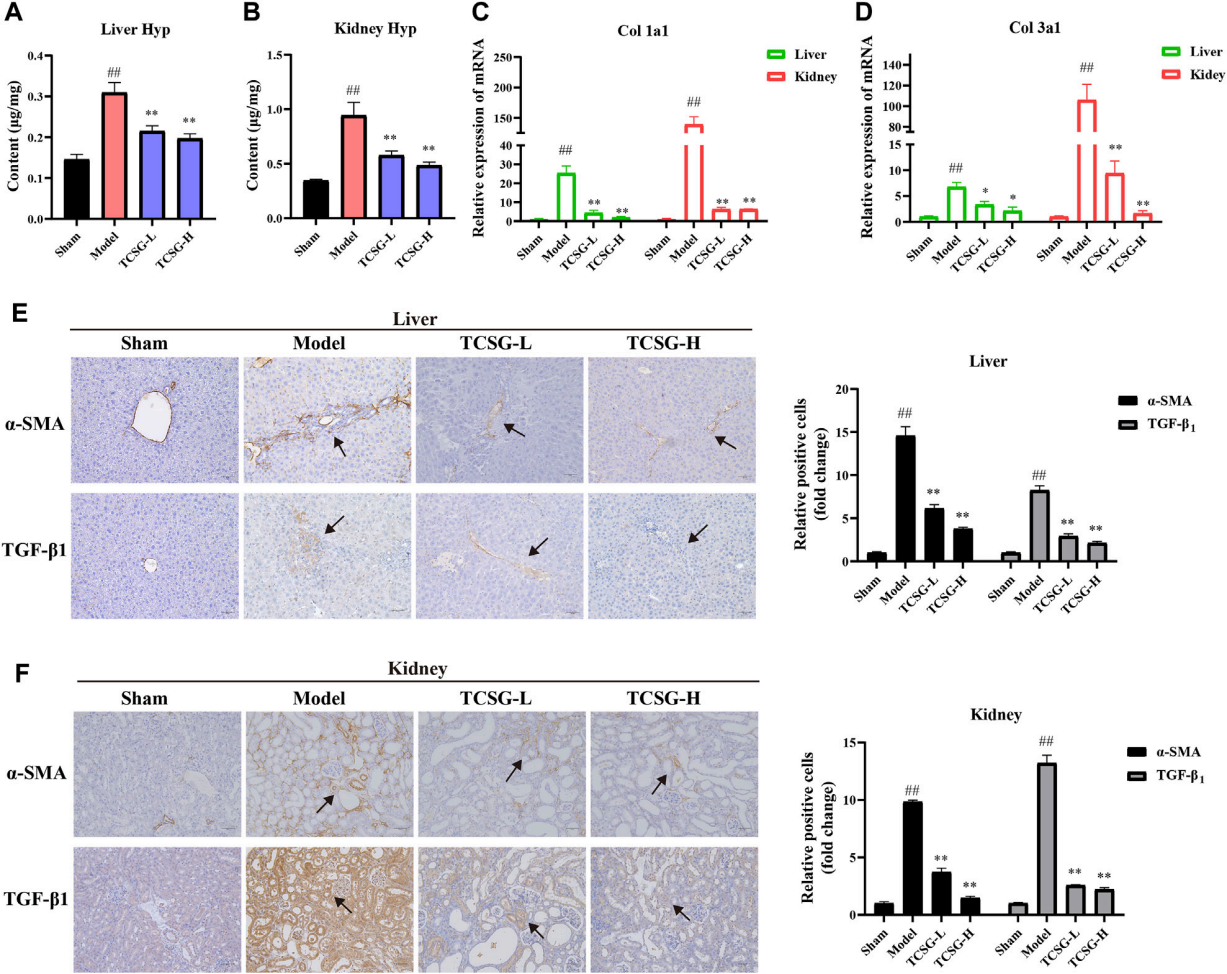
TCSG from *Baishouwu* inhibited hepatic and renal fibrosis in BDL&UUO mice. **(A)** Liver hydroxyproline concentration assays. **(B)** Kidney hydroxyproline concentration assays. **(C,D)** mRNA expressions of Col 1a1 and Col 3a1 in mice liver and kidney samples. **(E)** Immunohistochemistry and quantitative analysis of the levels of α-SMA and TGF-β1 in the liver. **(F)** Immunohistochemistry and quantitative analysis of the levels of α-SMA and TGF-β1 in the kidney. Data are shown as means ± SEM (*n* = 6), ^##^ *p* < 0.01, significantly different from sham group; ^*^ *p* < 0.05, ^**^ *p* < 0.01, significantly different from model group.

The visible collagen formation in the liver and kidney was associated with the progression of fibrosis, thus we further estimated the effects of TCSG on hepatic and renal fibrosis. The severity of fibrosis was determined biochemically by measuring Hyp content in the liver and kidney. Results showed the distinct upregulation of Hyp content in the liver and kidney of model mice, however, TCSG remarkably downregulated Hyp content (Figures 3A,B). The levels of fibrogenic markers in the liver and kidney, including Col 1a1 and Col 3a1, were significantly elevated in the model mice but decreased after treatment with TCSG (Figures 3C,D). Immunohistochemistry assays also validated that the expressions of TGF-β1 and α-SMA were increased in the liver and kidney of model mice, whereas TCSG treatment reduced the expression of these fibrogenic genes (Figures 3E,F). Accordingly, these data suggested that TCSG from *Baishouwu* exerted beneficial effects to inhibit the progression of hepatic fibrosis and renal fibrosis in BDL&UUO mice.

### Network Pharmacology Analyzed Potential Mechanisms for Antifibrotic Effects of TCSG From *Baishouwu*

**FIGURE 4:**
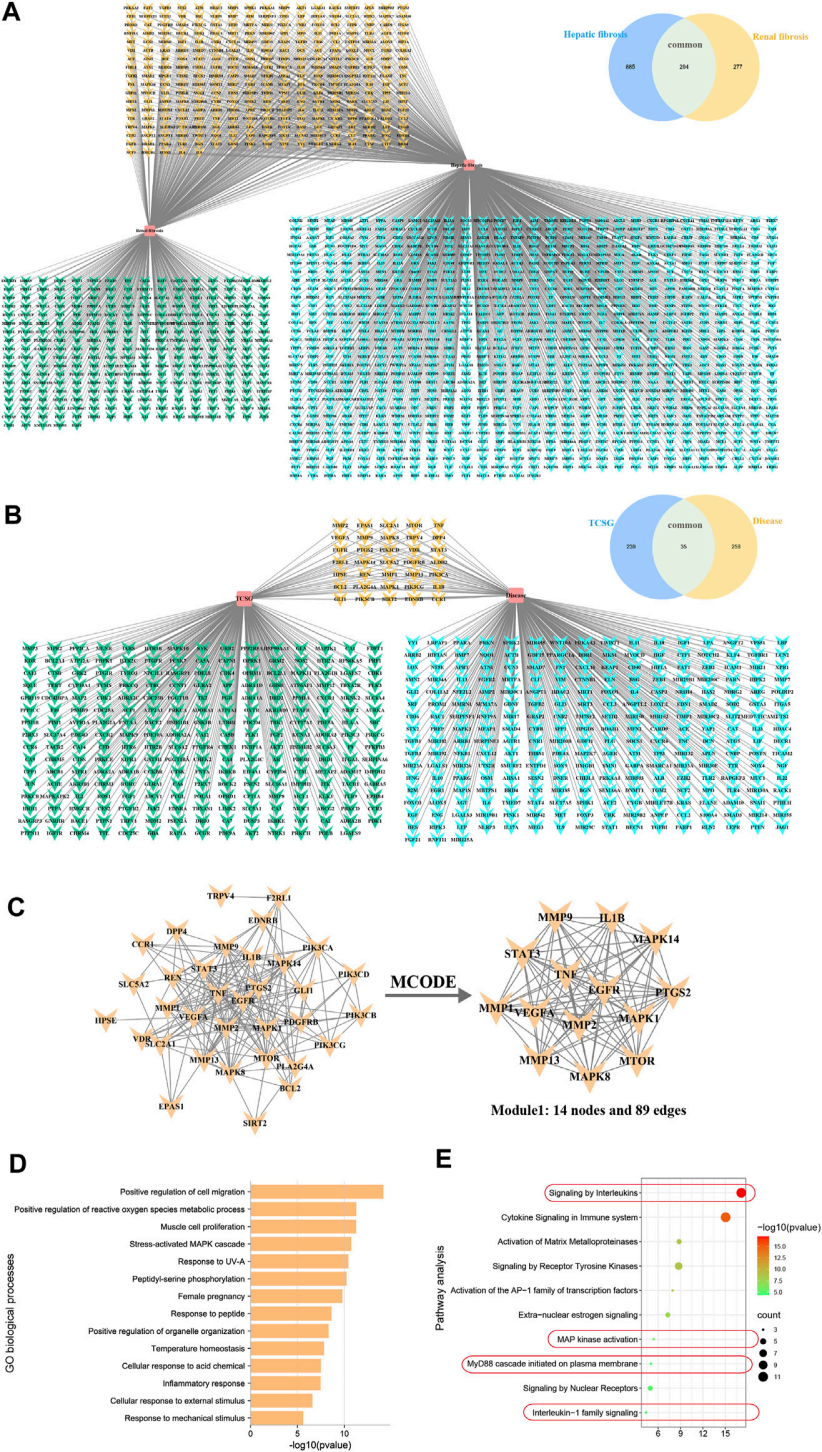
Network pharmacology analyzed potential mechanisms for antifibrotic effects of TCSG from *Baishouwu*. **(A)** Unique and shared disease targets for hepatic fibrosis and renal fibrosis. **(B)** Common targets of TCSG in treating hepatic and renal fibrosis. **(C)** Construction of PPI network for TCSG therapeutic targets and the core genes analyzed by plug-in MCODE. **(D)** GO enrichment analysis of core targets for TCSG in treating hepatic and renal fibrosis. **(E)** Reactome pathways enrichment analysis of core targets for TCSG in treating hepatic and renal fibrosis.

To explore the molecular mechanism associated with the anti-fibrotic effects of TCSG from *Baishouwu*, the network pharmacology technique was applied, which provided directions to illustrate the related potential targets and the hub for signaling pathways. As for 12 bioactive components in TCSG from *Baishouwu*, 274 targets were retrieved from databases and counted to construct the composition-target network (Supplementary Table S1 and Supplementary Figure S2). Meanwhile, a total of 1,456 targets were identified from 1,179 hepatic fibrosis-related targets and 571 renal fibrosis-related targets (Supplementary Table S2). Among them, 294 targets were shared by hepatic and renal fibrosis, which accounted for 24.94% of the hepatic fibrosis-related targets and 51.49% of the renal fibrosis-related targets (Figure 4A). The compound-disease network was constructed and 35 common targets were identified as potential therapeutic targets for TCSG against hepatic and renal fibrosis (Figure 4B). Then the PPI networks of potential therapeutic targets were built in the STRING database, which was subsequently imported in Cytoscape3.8.0 for analysis. The MCODE plug-in was used to calculate the modules involved in special functions. Module 1 got the best score of 13.69 and consisted of 14 hub target genes (Figure 4C). Furthermore, candidate targets of the core PPI network were subjected to GO biological processes and pathway enrichment analysis in the Reactome database. The relevant biological processes of TCSG against fibrosis of the liver and kidney were shown in Figure 4D and the significantly enriched pathways showed that the core targets were strongly associated with “Signaling by interleukins”, “MAP kinase activation”, “MyD88 cascade initiated on plasma membrane”, and “Interleukin-1 family signaling” (Figure 4E). Combined with the enrichment analysis results and the annotation of the Reactome database, it can be seen that TCSG from *Baishouwu* may affect inflammatory cascade through IL-1β/MyD88 signaling (Supplementary Figure S3).

### TCSG From *Baishouwu* Ameliorated Hepatic and Renal Fibrosis by Suppressing IL-1β/MyD88 Inflammation Signaling

**FIGURE 5:**
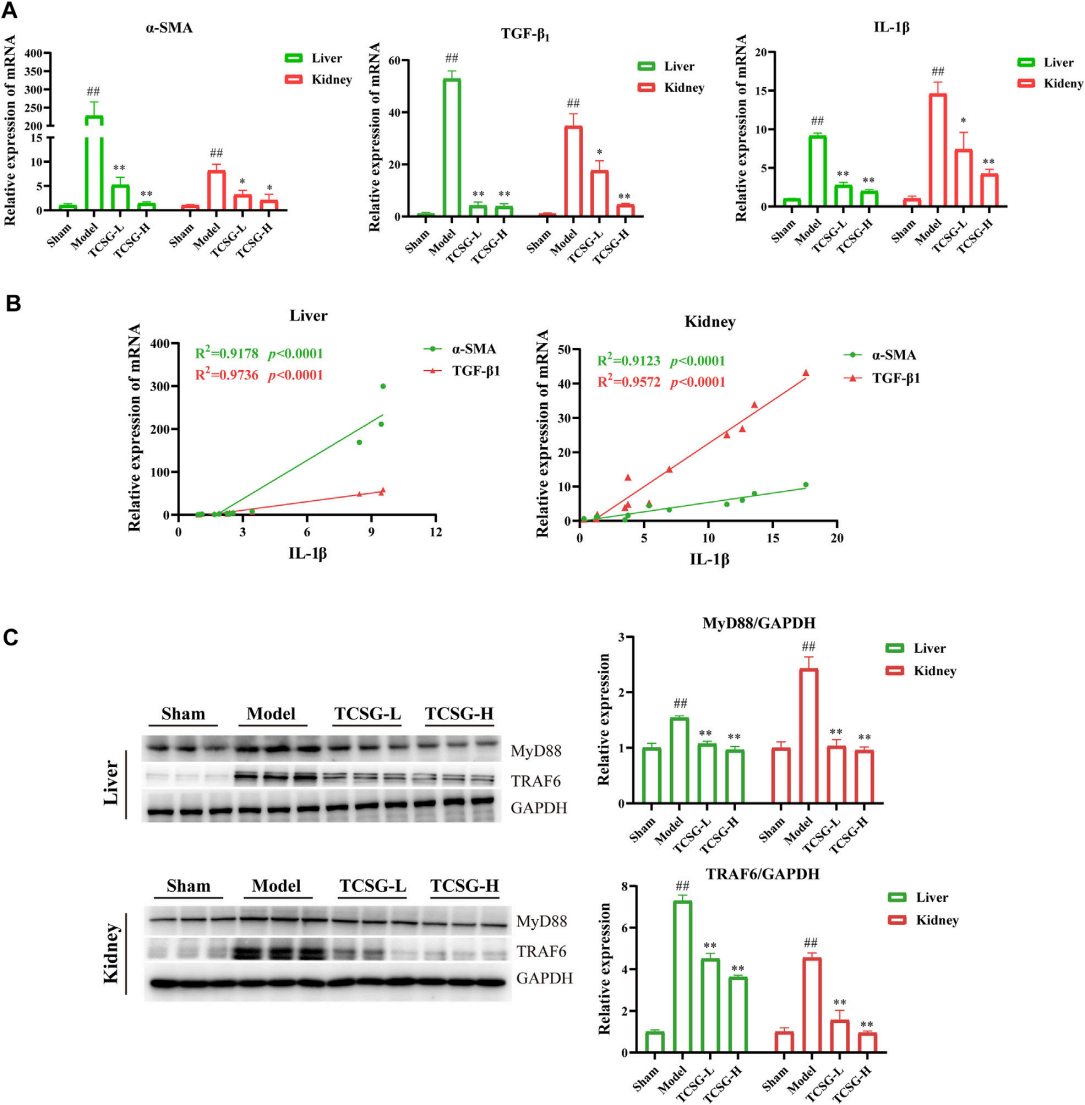
TCSG from *Baishouwu* ameliorated hepatic and renal fibrosis by suppressing IL-1β/MyD88 inflammation signaling. **(A)** mRNA expressions of α-SMA, TGF-β1, and IL-1β in the liver and kidney. **(B)** Correlation of IL-1β levels with α-SMA and TGF-β1 levels in the liver and kidney. R = Spearman’s rank correlation coefficient. **(C)** Western blot and quantitative analysis of MyD88 and TRAF6 expression in the liver and kidney. Data are shown as means ± SEM (*n* = 3), ^##^ *p* < 0.01, significantly different from sham group; ^*^ *p* < 0.05, ^**^ *p* < 0.01, significantly different from model group.

Although the signals driving fibrogenesis have not been fully elucidated, several lines of evidence suggest that IL-1 signals play a vital role in triggering fibrotic liver and kidney disease (Kamari et al., 2011; Lemos et al., 2018). According to the results of comprehensive informatics analysis, TCSG may exhibit the antifibrotic effect by affecting inflammatory cascade through IL-1β/MyD88 signaling. Thus, we validated the related mechanism in the BDL&UUO mice model. Results of qRT-PCR showed that the levels of α-SMA, TGF-β1, and IL-1β were significantly increased both in the liver and kidney of model mice, however, TCSG treatment reversed these changes (Figure 5A). Notably, regression analysis revealed that the levels of IL-1β were positively associated with the expression of α-SMA and TGF-β1 (Figures 5B,C), which suggested the possibility that TCSG-antifibrotic effects were tightly correlated with IL-1β signal transduction. Western bolt analysis displayed that the expression of MyD88 and TRAF6 was high, but was significantly reduced after treatment with TCSG in the liver and kidney of BDL&UUO mice (Figure 5D). To sum up, these data demonstrated that TCSG from *Baishouwu* could affect the IL-1β/MyD88 signaling in the BDL&UUO mice model, yet the downstream biological pathways needed further study.

### TCSG From *Baishouwu* Repressed p38 MAPK/JNK Signaling and Blocked the Nuclear Localization of NF-κB p65 in the Liver and Kidney

**FIGURE 6:**
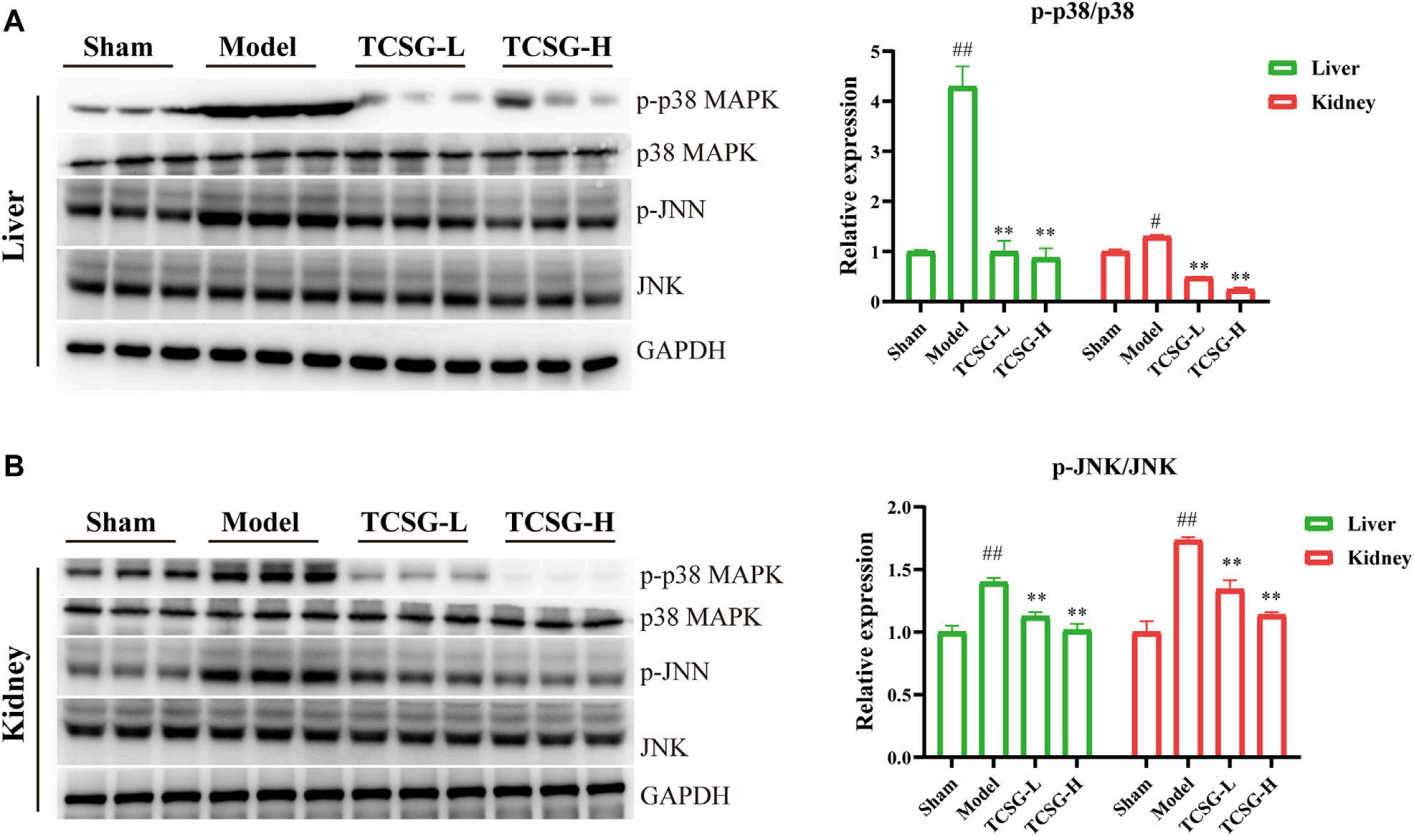
TCSG from *Baishouwu* repressed p38 MAPK/JNK signaling in the liver and kidney. **(A)** Western blot and quantitative analysis of p38 MAPK and p-JNK expression in the liver. **(B)** Western blot and quantitative analysis of p38 MAPK and p-JNK expression in the kidney. Data are shown as means ± SEM (*n* = 3), ^#^ *p* < 0.05, ^##^ *p* < 0.01, significantly different from sham group; ^**^ *p* < 0.01, significantly different from model group.

**FIGURE 7:**
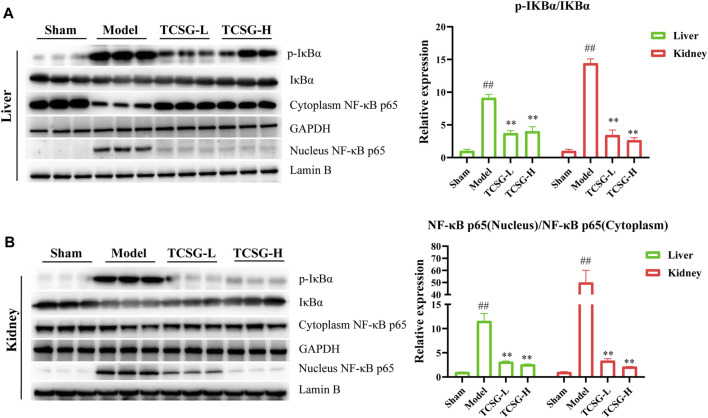
TCSG from *Baishouwu* blocked the nuclear localization of NF-κB p65 in the liver and kidney. **(A)** Western blot and quantitative analysis of p-IκBα and nucleus NF-κB p65 expression in the liver. **(B)** Western blot and quantitative analysis of p-IκBα and nucleus NF-κB p65 expression in the kidney. Data are shown as means ± SEM (*n* = 3), ^##^ *p* < 0.01, significantly different from sham group; ^**^ *p* < 0.01, significantly different from model group.

MAPK cascade and NF-κB family members act as essential components of the MyD88/TRIF6-dependent inflammation signal transduction pathway (Lim and Staudt, 2013; Liu and Ding, 2016). Results of enrichment analysis revealed that TCSG-related core targets were significantly enriched in MAP kinase (MAPK) activation and the hub target genes of TCSG against fibrosis contained MAPK14, one of the p38 MAPKs which plays an important role in response to inflammatory stimuli. Accordingly, we examined the effects of TCSG on p38 MAPK activation. As shown in Figure 6A, the levels of p-p38 MAPK and p-JNK were remarkably up-regulated in the liver of model mice compared to the control, whereas TCSG treatment alleviated these changes. Similarly, the levels of p-p38 MAPK and p-JNK were increased in the kidney of model mice but decreased after TCSG administration (Figure 6B). Furthermore, western blot results showed that TCSG treatment reduced the levels of p-IκB in the liver and kidney, the phosphorylation of which could subsequently liberate the active NF-κB, and the elevated levels of nuclear NF-κB p65 were also reversed by TCSG administration (Figures 7A,B). Together, these data demonstrated that p38 MAPK/JNK and NF-κB p65 signaling participated in antifibrotic effects of TCSG from *Baishouwu* in the BDL&UUO mice model.

## Discussion

In the current study, a mouse model of hepatic and renal fibrosis was employed to evaluate the protective effects of TCSG from *Baishouwu* on liver and kidney injury and to explore the correlation between hepatic and renal fibrosis. We found that TCSG from *Baishouwu* alleviated the dysfunction of the liver and kidney and reduced the degree of fibrosis both in the liver and kidney by inhibiting the generation of collagen in BDL&UUO mice. Network pharmacology analysis showed that inflammation response was a major contributing factor to the anti-fibrotic effects of TCSG from *Baishouwu* on the liver and kidney. Further studies demonstrated that TCSG from *Baishouwu* suppressed the development of hepatic and renal fibrosis through inhibiting MyD88 signal transduction activated by IL-1β and subsequent p38 MAPK and NF-κB p65 downstream signaling. Taken together, these findings suggest a potential correlation between hepatic and renal fibrosis and provide a new therapeutic approach to treat hepatic and renal fibrosis.

Emerging evidence reveals the crosstalk between organs which can affect their function. According to the clinical data, hepatic failure often leads to renal failure, and respectively, kidney dysfunction can result in dysregulated lipid metabolism and dyslipidemia (Wadei, 2012; Agrawal et al., 2018). As more studies demonstrate the strong correlation between chronic liver disease and kidney disease recently, there is an increased interest in understanding the liver-kidney axis (Raj et al., 2020; Heda et al., 2021). The previous study had developed a rodent model of BDL followed by lipopolysaccharide (LPS) injection to generate acute kidney injury and determine the pathophysiology of renal failure in cirrhosis, however, the development of renal fibrosis was not observed in the BDL&LPS model (Shah et al., 2012). We used to establish a model of hepatic and renal fibrosis by thioacetamide and UUO in SD rats, nevertheless, the low survival rate of the model group brought worry about the experimental animal amount (Zhuang et al., 2021). In the present study, we optimized the selection the coexistence of the hepatic and renal fibrosis model and explored the effects of medicine on the prevention and treatment of the disease. For the classical hepatic fibrosis model and renal interstitial fibrosis model, the combination of BDL and UUO can quickly establish a rodent model with the coexistence of hepatic and renal fibrosis. Our results showed that there was significant dysfunction of the liver and kidney in BDL&UUO mice, as well as well-characterized fibrosis both in the liver and kidney, which suggested that the BDL&UUO model could be employed to investigate the relationship between hepatic fibrosis and renal fibrosis.

*Baishouwu* is a traditional tonic that can boost the liver and kidney, enrich vital essence and blood, strengthen the bones and muscles, and benefit life essence based on the theory of TCM (Chen et al., 2019). According to the phytochemical studies, acetophenones and C-21 steroidal glycosides are the major bioactive constituents of *Baishouwu*. Moreover, it has been considered that the medicinal value of C-21 steroidal glycosides is more prominent. Pharmacological research reported that TCSG from *Baishouwu* could be used to treat chronic hepatitis and hepatic fibrosis and our previous study demonstrated that TCSG from *Baishouwu* had therapeutic effects on liver cancer and hepatic inflammation (Peng et al., 2008; Ding et al., 2019). In this study, we found that TCSG from *Baishouwu* remarkably improved the function of the liver and kidney and alleviated the degree of hepatic and renal fibrosis in BDL&UUO mice. Our data not only provided a potential therapeutic approach for hepatic and renal fibrosis therapy but also verified TCM theory “boost liver and kidney”. Furthermore, network pharmacological analysis was applied to assess the related biological system networks. Our previous research has determined the 12 bioactive components in TCSG from *Baishouwu* and the associated targets were obtained from the database. Finally, 35 corresponding targets were found to be therapeutic targets against hepatic and renal fibrosis, among which the most critical targets were IL-1β, MAPK14, PTGS2, mTOR, MAPK8, MMP13, MMP1, MMP9, MAPK1, STAT3, TNF, EGFR, VEGFA, and MMP2. The core targets were further subjected to function and pathway enrichment analysis, the result of which showed that TCSG might regulate inflammatory response through the cascade of events by Interleukins signaling, MAP kinase activation, MyD88 cascade initiated on plasma membrane, and Interleukin-1 family signaling pathways. In conclusion, these data provided the direction of underlying mechanisms for TCSG from *Baishouwu* against hepatic and renal fibrosis.

Chronic systemic inflammation is thought to have a pivotal role in the pathogenesis of hepatic fibrosis and renal fibrosis. It has become increasingly evident that excessive release of pro-inflammatory cytokines contributes to promoting the progression of fibrosis. In hepatic fibrosis, pro-inflammatory cytokines, including IL-1, IL-6, and TNF-α, generated by Kupffer, hepatocytes, and infiltrating inflammatory cells promote liver damage (Poniachik et al., 2006; Rock et al., 2010; Stienstra et al., 2010). Similarly, during kidney injury, pro-inflammatory cytokines trigger cell stress and drive secondary inflammatory mediators in the renal epithelium and interstitial stroma contributing to the renal fibrogenic process (Anders, 2014; Cao et al., 2015). More specifically, mounting evidence indicates that IL-1β can accelerate TGF-β1 expression and collagen production in the absence of Smad signaling (Cáceres et al., 2019), the reason of which lies in the fact that IL-1β exerts its effects by binding to IL-1 receptor and signal transduction adaptor MyD88, regulating fibrosis both in the liver and kidney (Kamari et al., 2011; Lemos et al., 2018). Our data showed the expression of α-SMA and TGF-β1 were positively correlated with the levels of IL-1β, indicating that the anti-fibrotic effects of TCSG from *Baishouwu* were closely related to IL-1β inflammatory response. Combined with the results of network pharmacological analysis, we further detected the MyD88 signaling and found that the current model of fibrogenesis activated MyD88/TRAF6 signal transduction in the liver and kidney, whereas TCSG from *Baishouwu* reversed the activation. This observation was consistent with previous studies pointing out that the IL-1β/MyD88-dependent mechanism could amplify inflammation and exacerbate fibrosis in the response to tissue injury (Gasse et al., 2007; Leaf et al., 2017). Our findings added further information in the understanding of inflammatory response in tissue injury and implicated the IL-1β/MyD88 signaling in the liver-kidney axis in the fibrogenic process.

The p38 MAPK and JNK signaling is an important intracellular signaling pathway associated with the production of profibrotic mediators. Inhibition of the activation of p38 MAPK or JNK can protect against inflammation and fibrosis (Sugiyama et al., 2012). Our study represented that TCSG from *Baishouwu* inhibited the phosphorylation of p38 MAPK and JNK induced by BDL&UUO surgery in the liver and kidney, indicating that p38 MAPK/JNK signaling was a downstream effector signal of inflammatory response affected by TCSG from *Baishouwu*. In addition, we have observed that TCSG from *Baishouwu* has affected the activation of NF-κB p65, which is a key transcriptional regulator of inflammatory signals and appears to regulate fibrosis via multiple mechanisms, including direct fibrogenic responses and antiapoptotic effects (Luedde and Schwabe, 2011). In the canonical pathway, IκBα is the most prominent inhibitor of NF-κB p65 activity, the phosphorylation of which can subsequently be degraded and liberates NF-κB p65 for nuclear entry. The current study demonstrated that inhibition of the activation of p38 MAPK/JNK and NF-κB p65 signaling cascade exerted protective effects against hepatic and renal fibrosis, nevertheless, previous research had pointed out that NF-κB p65 could collaborate with p38 MAPK and resist hepatocyte apoptosis induced by TNF or LPS (Heinrichsdorff et al., 2008). These contradictory conclusions show an interesting finding that NF-κB mediates both pro-inflammatory and anti-apoptotic responses among different models of liver injury, and a very weak or a very strong NF-κB activation may cause negative effects on the liver. Complete blockage of TNF-induced NF-κB activation in conditional deletion animals resulted in massive hepatocyte apoptosis after LPS injection (Luedde et al., 2007; Inokuchi et al., 2010), while inhibition of NF-κB in Kupffer cells or hepatic stellate cells led to a decrease in the severity of liver fibrosis both in chronically injured animals and patients with hepatitis C virus (Son et al., 2007; Oakley et al., 2009). It is noted that excessive stimulation of IL-1β promoted NF-κB activation to exceed its threshold and thereby accentuated hepatic inflammation and fibrosis in the BDL&UUO mice model. Accordingly, NF-κB p65 signaling was also a downstream effector of TCSG against fibrosis.

Although the current data suggested the role of IL-1β/MyD88 inflammation signaling in the liver-kidney axis to a certain extent, there are still some limitations to be further solved. Whether the pathological changes in the model of BDL&UUO were the direct toxicity of organs at the same time or the injury based on the interaction of liver and kidney function was unclear. As a result, it needs to systematically explore the changes in the model of BDL&UUO using omics technology in future research. Moreover, the establishment of co-culture of hepatic stellate cells and renal tubular epithelial cells to examine the relationship of liver and kidney under the influence of inflammatory factors can also contribute to enhancing the understanding of the crosstalk between the liver and kidney.

## Conclusion

**FIGURE 8:**
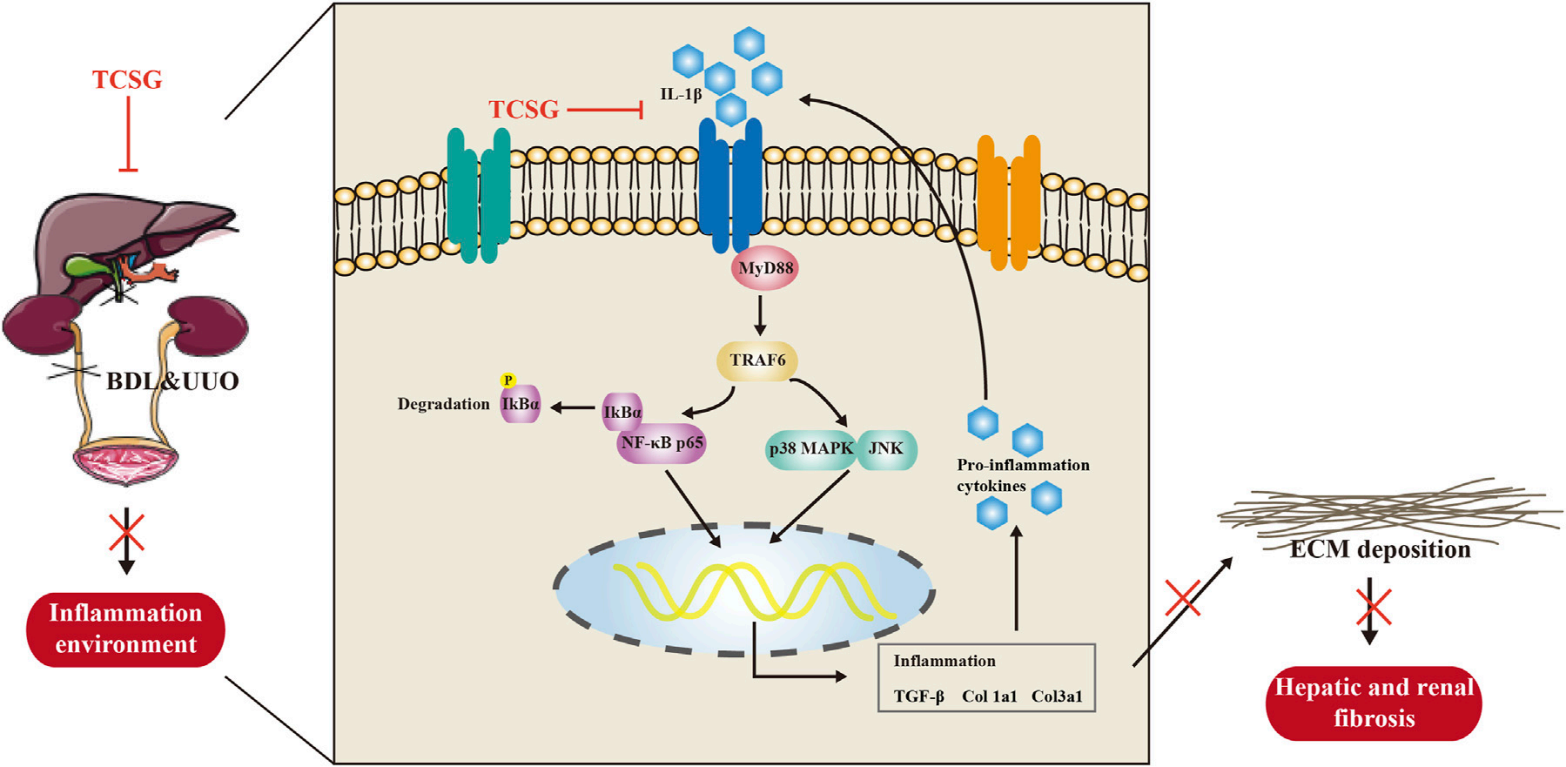
The schematic diagram of the potential mechanism of TCSG from Baishouwu against hepatic and renal fibrosis.

Our results demonstrate strong evidence for the anti-inflammatory effect of TCSG from *Baishouwu* on hepatic and renal fibrosis in the BDL&UUO mice model. The present study highlights the importance of the IL-1β/MyD88-induced inflammation signaling in the development of hepatic and renal fibrosis (Figure 8). The insights obtained from this study have important implications for the development of novel therapies for hepatic and renal fibrosis.

## Data Availability

The original contributions presented in the study are included in the article/Supplementary Material, further inquiries can be directed to the corresponding authors.
