# Supplementary material for: Total C-21 Steroidal Glycosides From Baishouwu Ameliorate Hepatic and Renal Fibrosis by Regulating IL-1β/MyD88 Inflammation Signaling
Source: Front Pharmacol. 2021 Oct 26;12:775730. doi: 10.3389/fphar.2021.775730 (PMC8576092; doi:10.3389/fphar.2021.775730)
Supplement: Supplementary file 1 [file DataSheet2.PDF]

Figure 5C

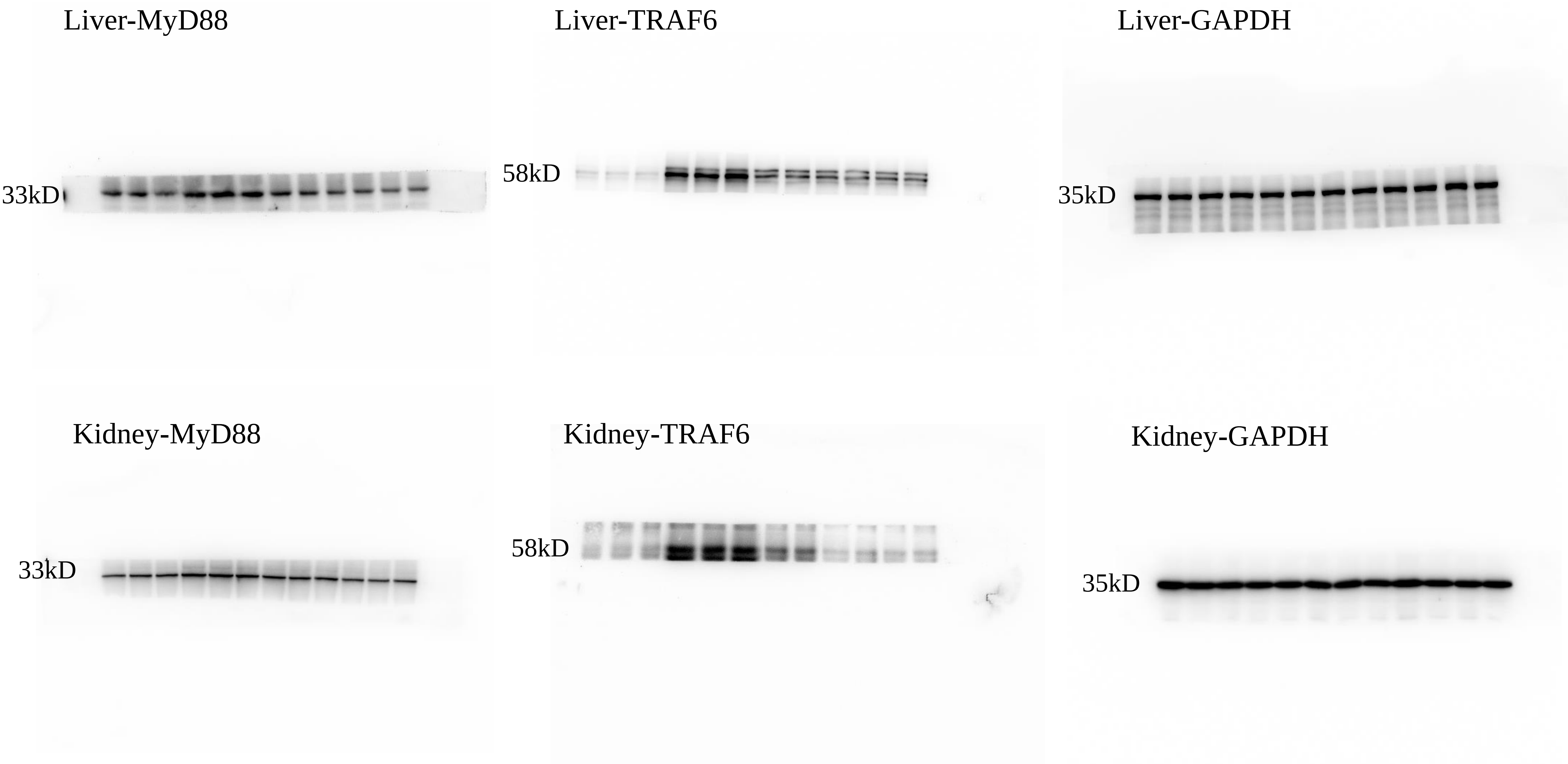
Liver-MyD88
33kD
Liver-TRAF6
58kD
Liver-GAPDH
35kD
Kidney-MyD88
33kD
Kidney-TRAF6
58kD
Kidney-GAPDH
35kD

Figure 6

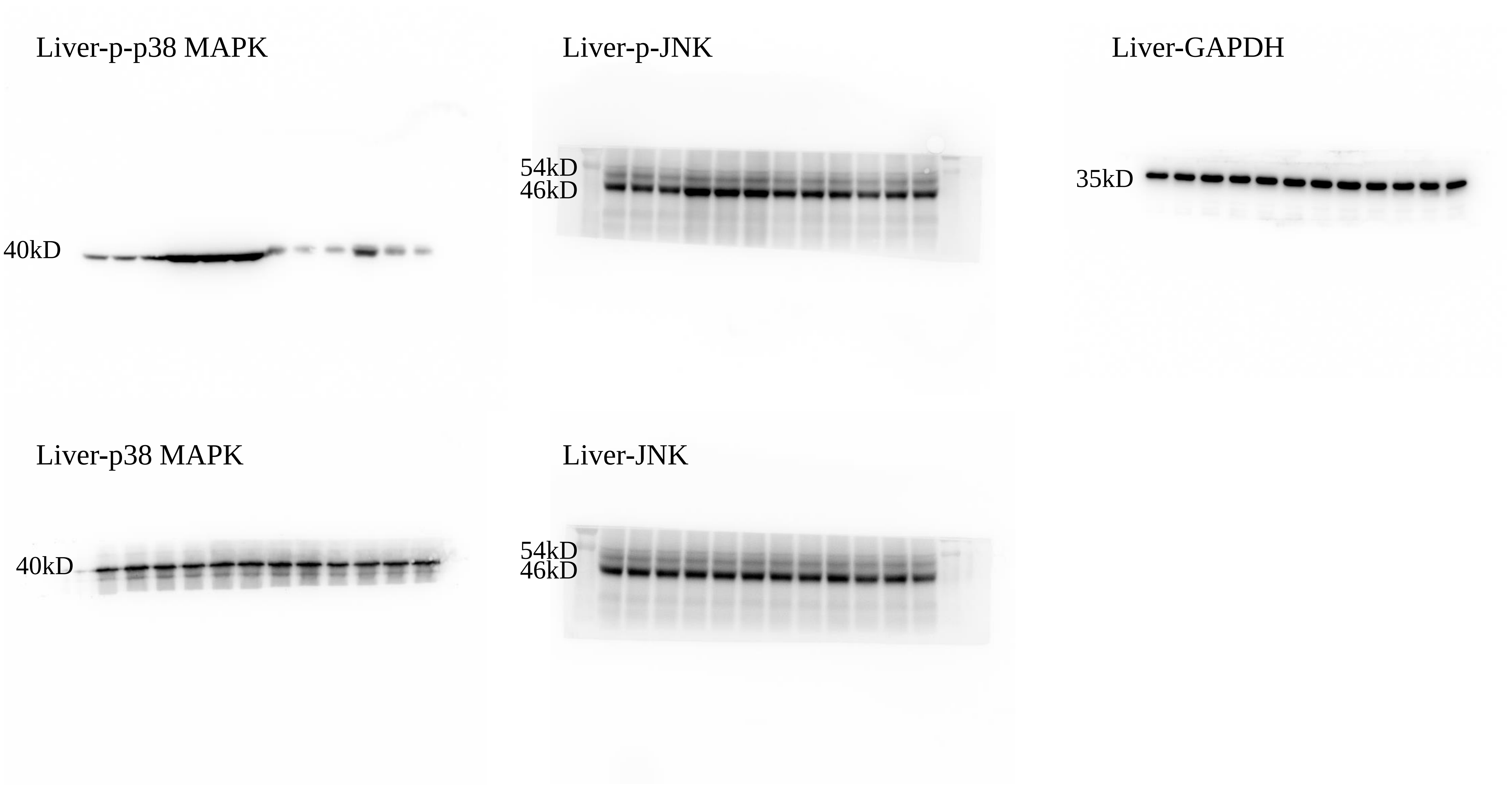
Liver-p-p38 MAPK
40kD
Liver-p-JNK
54kD
46kD
Liver-GAPDH
35kD
Liver-p38 MAPK
40kD
Liver-JNK
54kD
46kD

Figure 6

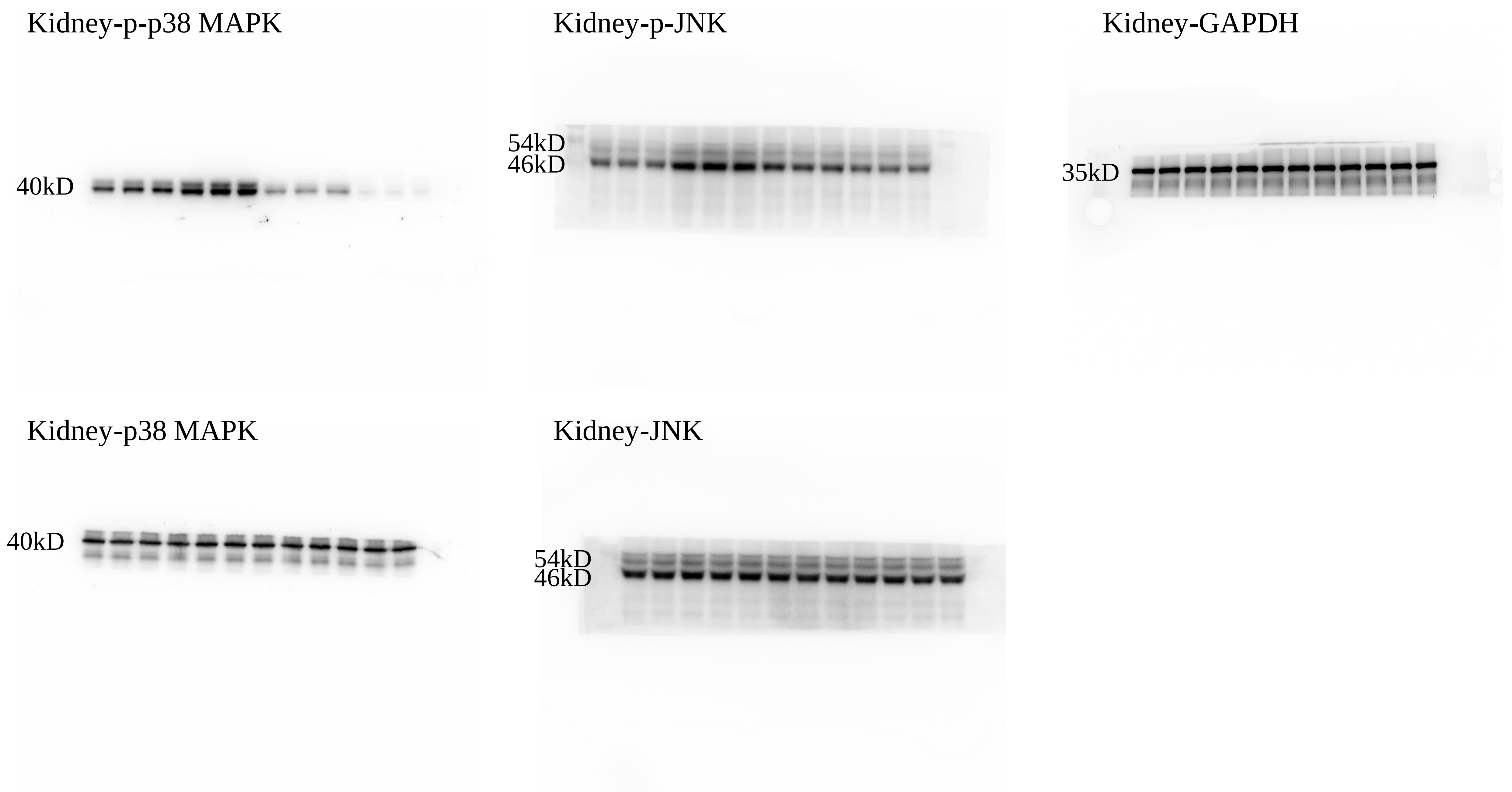
Kidney-p-p38 MAPK
40kD
Kidney-p-JNK
54kD
46kD
Kidney-GAPDH
35kD
Kidney-p38 MAPK
40kD
Kidney-JNK
54kD
46kD

Figure 7

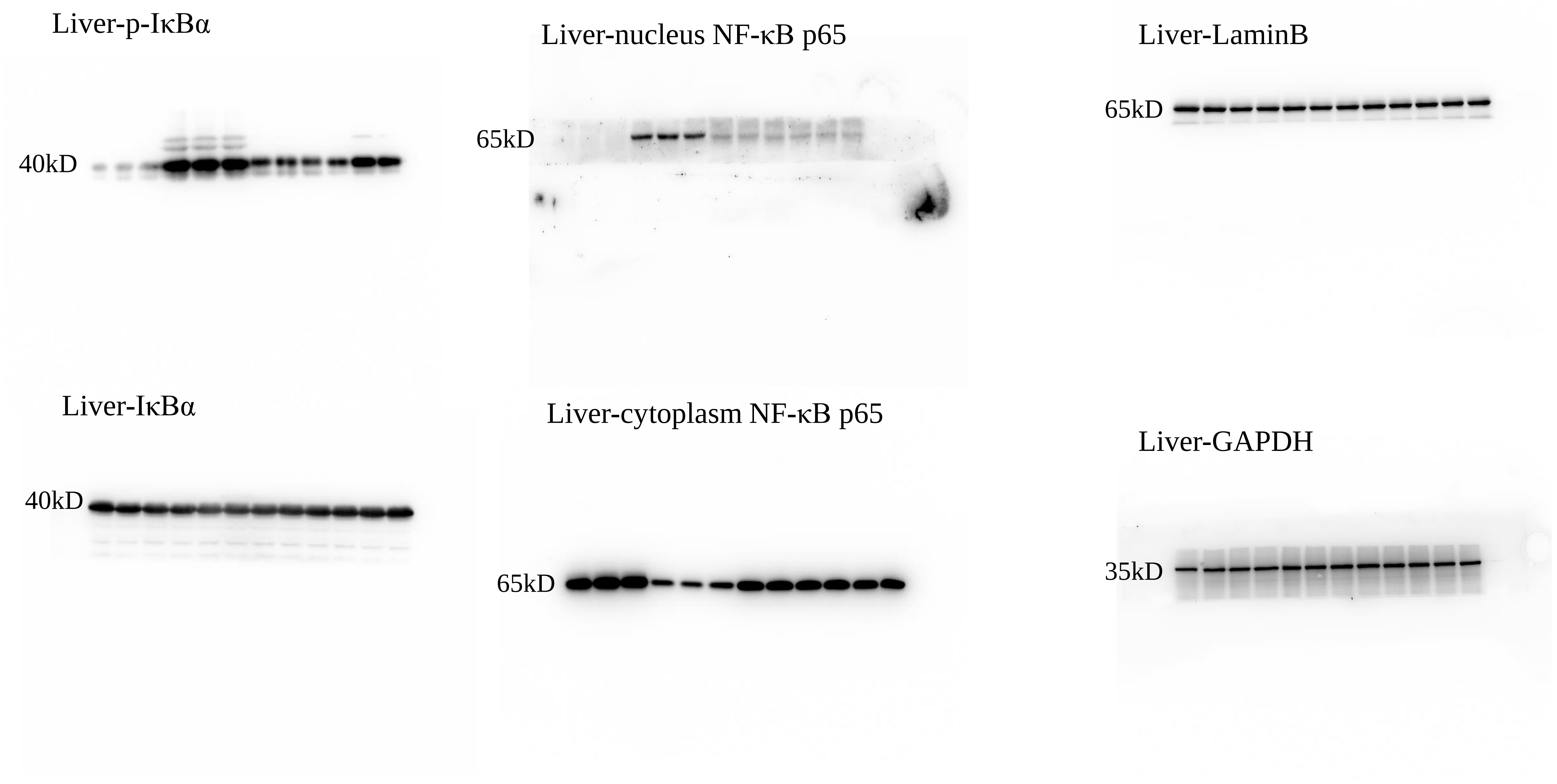
Liver-p-IκBα
40kD
Liver-nucleus NF-κB p65
65kD
Liver-LaminB
65kD
Liver-IκBα
40kD
Liver-cytoplasm NF-κB p65
65kD
Liver-GAPDH
35kD

Figure 7

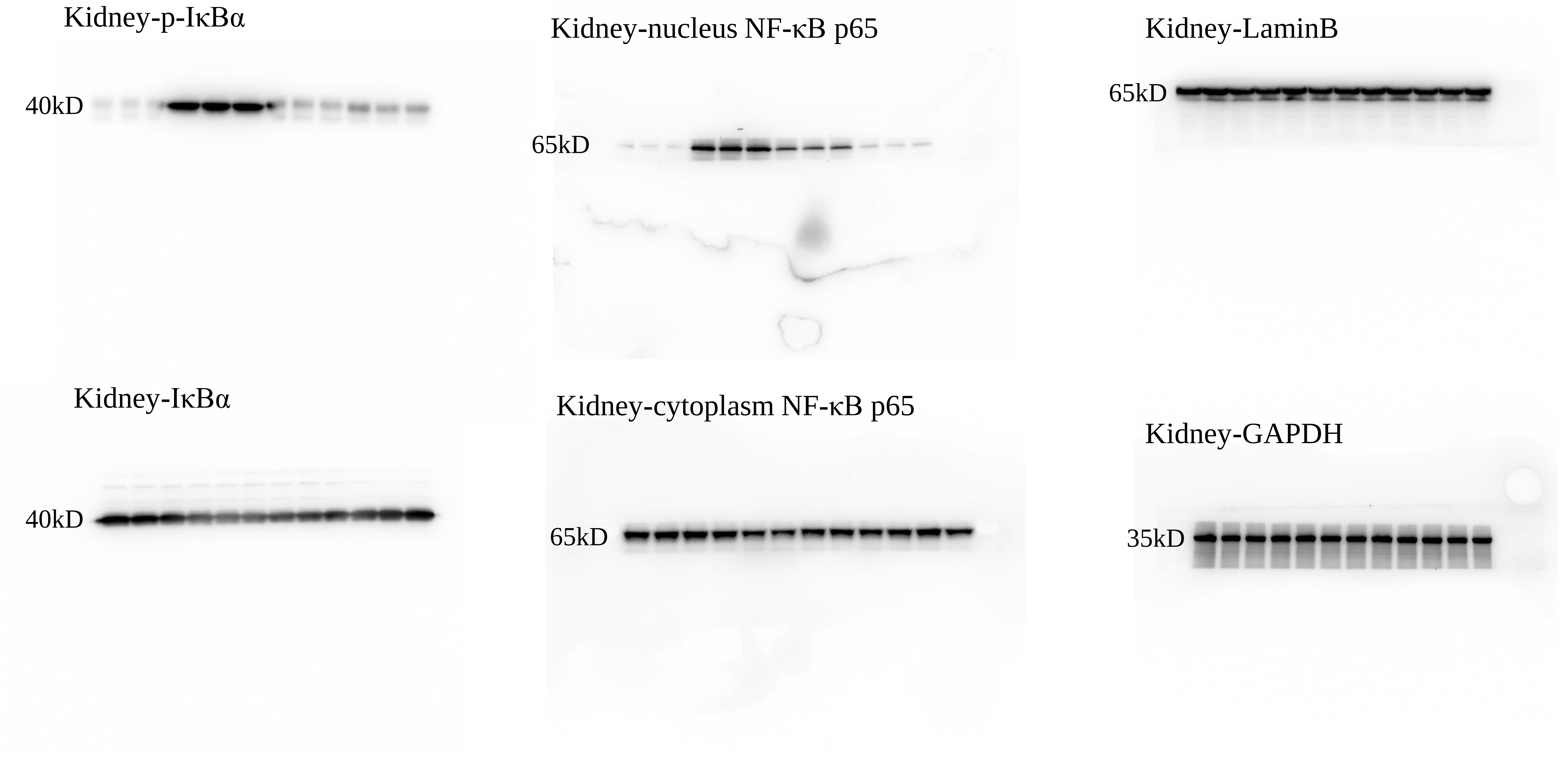

Kidney-p-IκBα
40kD
Kidney-nucleus NF-κB p65
65kD
Kidney-LaminB
65kD
Kidney-IκBα
40kD
Kidney-cytoplasm NF-κB p65
65kD
Kidney-GAPDH
35kD
