# Supplementary figures and images for: Total C-21 Steroidal Glycosides From Baishouwu Ameliorate Hepatic and Renal Fibrosis by Regulating IL-1β/MyD88 Inflammation Signaling
Source: Front Pharmacol. 2021 Oct 26;12:775730. doi: 10.3389/fphar.2021.775730 (PMC8576092; doi:10.3389/fphar.2021.775730)

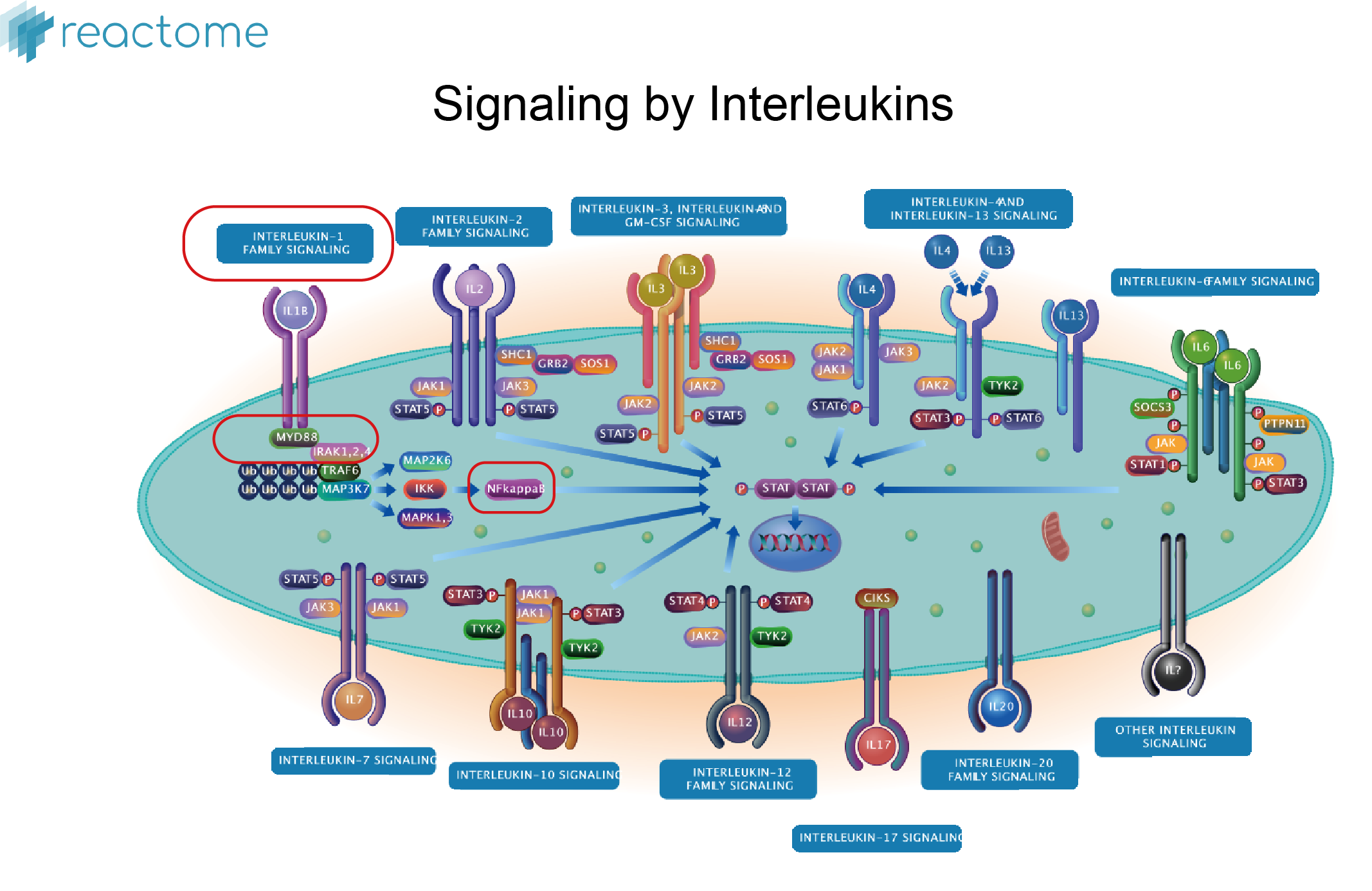

Supplement: Supplementary file 3 [file Image3.TIF]

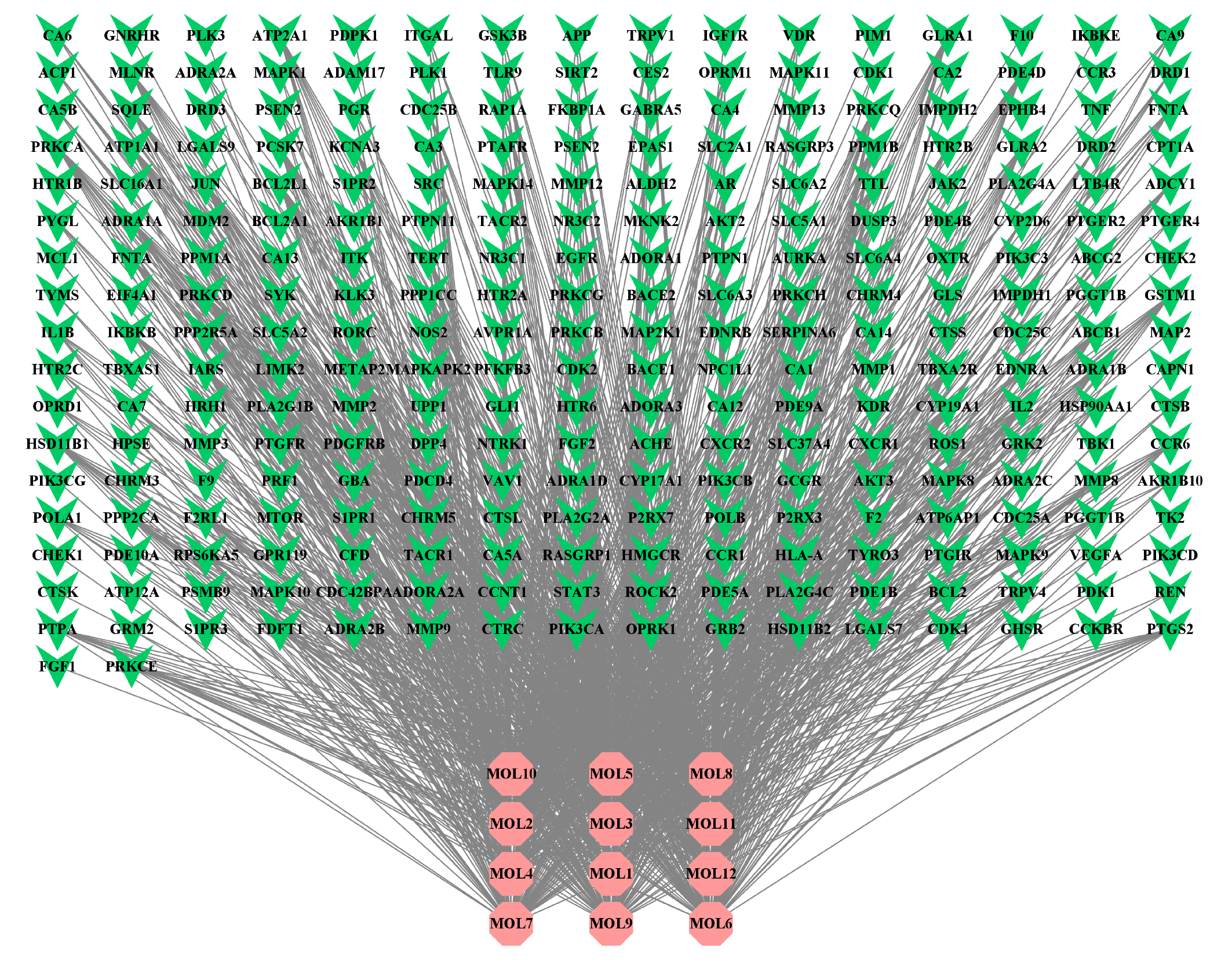

Supplement: Supplementary file 4 [file Image2.TIF]

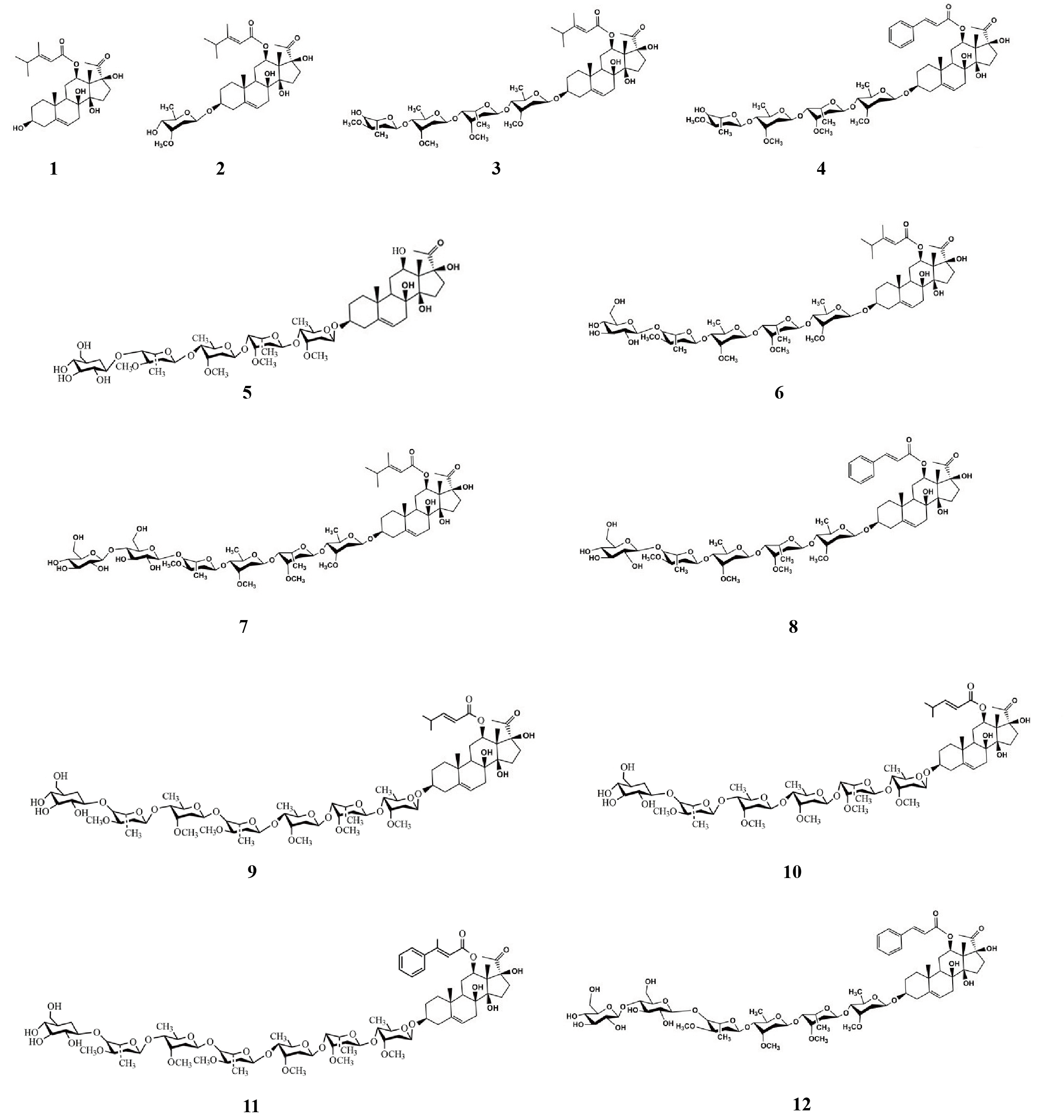

Supplement: Supplementary file 5 [file Image1.TIF]
