# Supplementary material for: Total C-21 Steroidal Glycosides From Baishouwu Ameliorate Hepatic and Renal Fibrosis by Regulating IL-1β/MyD88 Inflammation Signaling
Source: Front Pharmacol. 2021 Oct 26;12:775730. doi: 10.3389/fphar.2021.775730 (PMC8576092; doi:10.3389/fphar.2021.775730)
Supplement: Supplementary file 8 [file DataSheet1.docx]

**Total C-21 steroidal glycosides from *Baishouwu* ameliorate hepatic and renal fibrosis by regulating IL-1β/MyD88 inflammation signaling**

Tingting Qin^1, 2, #^, Mingliang Wang^1, 2, #^, Ting Zhang^1, 2^, Yingyu Wang^1,2^, Yunyun Zhang^1, 2^, Muhammad Hasnat^3^, Zirui Zhuang^1, 2^, Yongfang Ding^1, 2, *^, Yunru Peng^1, 2, *^

1 Affiliated Hospital of Integrated Traditional Chinese and Western Medicine, Nanjing University of Chinese Medicine, Nanjing 210028, China

2 Department of Pharmacology and Toxicology, Jiangsu Province Academy of Traditional Chinese Medicine, Nanjing 210028, China

3 Institute of Pharmaceutical Sciences, University of Veterinary and Animal Sciences, Lahore 54600, Punjab, Pakistan

* Corresponding author:

Professor Yunru Peng, PhD

Professor Yongfang Ding, PhD

Tel: +86 52362105, E-mail: pengyunru@126.com (Yunru Peng); Yongfangding@gmail.com (Yongfang Ding)

# These authors have contributed equally to this work.

Figure S1 The details of 12 active ingredients in TCSG from *Baishouwu*.

Figure S2 The network of targets for active ingredients in TCSG from *Baishouwu*.

Figure S3 The annotation of signaling by interleukins from Reactome Database.

Table S1 The potential targets of active ingredients in TCSG from *Baishouwu*.

Table S2 The known targets of hepatic fibrosis and renal fibrosis from the DisGeNET Database.
